# Association of Serum Gamma-Glutamyl Transferase and Ferritin with the Metabolic Syndrome

**DOI:** 10.1155/2015/741731

**Published:** 2015-06-21

**Authors:** Dong Wei, Tao Chen, Jie Li, Yun Gao, Yan Ren, Xiangxun Zhang, Hongling Yu, Haoming Tian

**Affiliations:** ^1^Department of Endocrinology and Metabolism, West China Hospital of Sichuan University, Chengdu 610041, China; ^2^Department of Endocrinology and Metabolism, The Second People's Hospital of Chengdu, Chengdu 610017, China; ^3^Department of Pediatrics, University of Pittsburgh School of Medicine, Pittsburgh, PA 15224, USA; ^4^Laboratory of Endocrinology and Metabolism, West China Hospital of Sichuan University, Chengdu 610041, China

## Abstract

*Aim*. To investigate the relationship among GGT, ferritin, and the risk of metabolic syndrome. *Methods*. A total of 1024 eligible individuals of the Chinese Yi ethnic group were enrolled in this cross-sectional study. The presence of metabolic syndrome was determined using the revised NCEP-ATP III and CDS criteria. Odds ratios for the metabolic syndrome and its components for different groups based on the levels of GGT and ferritin were calculated using multiple logistic regressions. *Results*. Serum GGT and ferritin concentrations were significantly higher in subjects with metabolic syndrome compared to those without metabolic syndrome in both genders (*p* < 0.05). Serum GGT was positively correlated with ferritin (*p* < 0.05). The risk of the metabolic syndrome was significantly higher in female subjects who had elevated GGT and ferritin levels (*p* < 0.05). Furthermore, the increased risk of having each of the metabolic syndrome components (overweight or obesity, hypertriglyceridemia, hypertension, hyperglycemia, and insulin resistance) was also observed in those subjects after adjustment for possible confounders (*p* < 0.05). *Conclusions*. These data indicate that GGT and ferritin synergistically correlate with the risk of the metabolic syndrome, suggesting that they could potentially be used as predictive biomarkers for the metabolic syndrome.

## 1. Introduction

The metabolic syndrome is a cluster of interrelated metabolic abnormalities that directly promote the development of atherosclerotic cardiovascular disease. Its main components are obesity, dyslipidemia, hypertension, insulin resistance, and abnormal glucose tolerance. Currently, there is not a standard definition of the metabolic syndrome that spans different populations, although some criteria have been proposed by various organizations to address this issue [1–5]. Insulin resistance is generally considered to be the central component that is involved in the common pathogenesis of the metabolic syndrome.

Gamma-glutamyl transferase (GGT) and ferritin participate in common pathophysiological processes, including oxidative stress and lipid peroxidation, which are important to the pathogenesis and development of insulin resistance and the metabolic syndrome [6–8]. A number of studies have shown that the serum level of GGT directly correlates with increased risk of death, hypertension, diabetes, and metabolic syndrome, even after adjustment for alcohol consumption and established risk factors [6–11]. In addition, several studies have shown that a high level of serum ferritin, a sensitive indicator of body iron stores, is associated independently with an increased risk of type 2 diabetes and the metabolic syndrome [12–14]. However, conflicts exist among these studies with regard to these associations among different ethnicities/races, gender, and alcohol consumption status. Furthermore, most of these studies focused specifically on either GGT or ferritin rather than addressing the synergistic association of GGT and ferritin with the metabolic syndrome.

Previously, our group conducted a novel investigation in a Chinese ethnic minority cohort of the synergistic association between GGT and ferritin and the risk of developing type 2 diabetes, an individual component of the metabolic syndrome [15]. We confirmed the synergistic association of GGT and ferritin with type 2 diabetes in women and suggested oxidative stress and lipid peroxidation as potential mechanisms. In this study, we investigated the relationship between these two metabolic markers and the metabolic syndrome in both genders.

## 2. Materials and Methods

### 2.1. Design

The study was part of the China National Diabetes and Metabolic Disorders Study, a population-based cross-sectional study designed to investigate the prevalence of type 2 diabetes and metabolic syndrome in the Chinese population [16]. A total of 1288 individuals of the Yi ethnic group from Xichang of Liangshan Yi Nationality Autonomous District, Sichuan Province of China, participated in this survey. This study was approved by China-Japan Friendship Hospital's Drugs/Medical Apparatus & Instruments Ethics Committee and was registered on the website of the Chinese Clinical Trial Registry (ChiCTR-OCS-09000361). Written informed consent was obtained from each participant before data collection. This study was carried out in compliance with the Helsinki Declaration II.

### 2.2. Measurements and Populations

A standard questionnaire collecting demographic characteristics, medical history, and lifestyle factors was provided to the subjects. The medical history included the diagnosis and treatment of diabetes, hypertension, dyslipidemia, cardiovascular events, liver diseases, and malignant diseases. Those with a known history of liver disease (e.g., acute and chronic active hepatitis, liver cirrhosis), biliary tract diseases, malignant diseases, acute infectious or inflammatory disorders, history of transfusion, and iron therapy were all excluded. Lifestyle factors assessed by the questionnaire included alcohol consumption, smoking habits, and recreational physical activity. Heavy smoking and heavy drinking were defined as exposure to more than 25 cigarettes per day and consumption of more than 60 mL of alcohol per day, respectively [15]. Regular recreational physical activity was defined as participation in moderate or vigorous activity for at least 30 minutes or longer per day at least 3 days a week [17].

Blood pressure (BP) was measured by standard methods. Body weight, height, and waist circumference were obtained. Body mass index (BMI) was calculated as weight in kilograms divided by the square of height in meters (kg/m^2^). Blood samples were taken at the time of enrollment. The participants were asked to fast for at least 8 hours before their examinations. A standard 75 g glucose solution or a steamed bun that contained approximately 80 g of complex carbohydrates was provided as oral glucose tolerance test (OGTT). Plasma glucose was measured by a hexokinase enzymatic method. Serum ferritin was measured with a radioimmunoassay kit (Beijing North Institute of Biological Technology). Alanine aminotransferase (ALT), aspartate aminotransferase (AST), and GGT were determined using enzymatic methods. Serum lipids, uric acid (UA), hepatitis B surface antigen (HBsAg), and anti-hepatitis C virus (anti-HCV) as well as urinary albumin-to-creatinine ratio (UACR) were determined according to standard laboratory procedures. All measurements were conducted within 2 hours of sample collection. Insulin resistance was examined by the homeostatic model of assessment for insulin resistance (HOMA-IR). The HOMA-IR index was calculated using the following formula: fasting serum insulin (mU/mL) × fasting plasma glucose (mmol/L)/22.5. A HOMA-IR index score above the upper quartile of normal glucose tolerance (NGT) populations was used to determine the presence of insulin resistance according to World Health Organization (WHO) criteria [1].

The presence of metabolic syndrome was determined using two definitions: one proposed by the revised National Cholesterol Education Program Expert Panel on Detection, Evaluation and Treatment of High Blood Cholesterol in Adults Adult Treatment Panel III (NCEP-ATP III) and one proposed by the China Diabetes Society (CDS) [3, 5]. According to the revised NCEP ATP-III criteria, a participant has the metabolic syndrome if he or she meets three or more of the following criteria: (1) abdominal obesity (waist circumference ≥ 90 cm in men, ≥80 cm in women); (2) hypertriglyceridemia (triglycerides (TG) ≥ 1.70 mmol/L or on drug treatment for elevated TG); (3) low high density lipoprotein-cholesterol (HDL-C) (HDL-C < 1.03 mmol/L in men, <1.3 mmol/L in women, or on drug treatment for reduced HDL-C); (4) hypertension (systolic blood pressure (SP) ≥ 130 mmHg, diastolic pressure (DP) ≥ 85 mmHg, or on antihypertensive medication); (5) high fasting glucose (fasting plasma glucose (FPG) ≥ 5.6 mmol/L or under treatment for diabetes). The CDS definition of the metabolic syndrome requires three or more abnormalities of the following criteria: (1) overweight or obesity (BMI ≥ 25.0 kg/m^2^); (2) dyslipidemia (TG ≥ 1.70 mmol/L and/or low HDL-cholesterol [<0.9 mmol/L in men, <1.0 mmol/L in women]); (3) hypertension (SP ≥ 140 mmHg, DP ≥ 90 mmHg, or on antihypertensive medication); (4) hyperglycemia (FPG ≥ 6.1 mmol/L and/or 2-hour postprandial plasma glucose (PG) ≥ 7.8 mmol/L, or under treatment for diabetes).

### 2.3. Statistical Analysis

Data were presented as mean (standard deviation) or medians (interquartile range) for continuous data and as numbers (in percentage) for categorical measures. Data that were not normally distributed were logarithmically transformed for analysis. Student's *t*-test, Mann-Whitney *U* test, or Chi-square test was used to compare the difference of clinical and laboratory characteristics between the subjects with and without the metabolic syndrome. Spearman's rank-order correlation coefficient and multiple linear regression analyses were applied to determine the relationship between GGT and ferritin. Participants were divided into three groups based on the levels of GGT and ferritin: Group 1 including subjects with both GGT and ferritin lower than median values, Group 2 with only GGT or ferritin higher than median values, and Group 3 with both GGT and ferritin higher than median values. Differences of clinical and laboratory data among groups were calculated using one-way analysis of variance or Kruskal-Wallis rank-sum test. Odds ratios (ORs) for the metabolic syndrome and its components in Group 2 and Group 3 compared with Group 1 were analyzed by multiple logistic regressions. Statistical analysis was performed using SPSS (version 15.1; SPSS, Inc., Chicago, IL). *p* value of less than 0.05 was considered to be statistically significant.

## 3. Results

### 3.1. Clinical and Laboratory Characteristics of Subjects

A total of 1288 individuals, aged 20–74 years, participated in this survey. 1024 individuals (84.0%), including 436 men and 588 women, were eligible subjects whose data proceeded to final analysis. Clinical and laboratory characteristics of the study participants are presented in Table 1. In total, 197 (19.2%) subjects were identified as having metabolic syndrome and 827 (80.8%) as not having metabolic syndrome according to the revised NCEP-ATP III criteria; 135 (13.2%) subjects were identified as having metabolic syndrome and 889 (86.8%) as not having metabolic syndrome according to the CDS criteria. In female participants, we identified 161 (27.4%) using the revised NCEP-ATP III definition and 79 (13.4%) using the CDS definition as having metabolic syndrome. The numbers and percentages of the metabolic syndrome in male subjects were 36 (8.3%) according to the revised NCEP-ATP III definition and 56 (12.8%) according to the CDS definition, respectively (Table 1). Serum GGT and ferritin levels were significantly higher in subjects with metabolic syndrome compared to those without metabolic syndrome in both genders. (Serum GGT: *p* = 0.000 according to the revised NCEP-ATP III and CDS definitions in females; *p* = 0.003 according the revised NCEP-ATP III definition and *p* = 0.000 according the CDS definition in males. Serum ferritin: *p* = 0.000 according to the revised NCEP-ATP III and CDS definitions in females; *p* = 0.003 according the revised NCEP-ATP III definition and *p* = 0.000 according the CDS definition in males.) In both genders, BMI, waist circumference, SP, DP, TG, 2h PG, and HOMA-IR were significantly higher in subjects with metabolic syndrome compared to those without (*p* < 0.05). FPG was significantly higher in the metabolic syndrome in female subjects (*p* < 0.05) but not in males. There was no significant difference in HDL-C level in subjects with or without metabolic syndrome in females (*p* > 0.05) (Table 1).

### 3.2. Association between GGT and Ferritin

Spearman correlation analysis showed that GGT was significantly correlated with ferritin (*r* = 0.425, *p* = 0.000), and both of them were significantly correlated with gender (*r* = −0.499, *p* = 0.000 for ferritin; *r* = −0.277, *p* = 0.000 for GGT). Spearman correlation analysis confirmed that GGT was significantly correlated with ferritin in both genders (*r* = 0.256, *p* = 0.000 for females; *r* = 0.437, *p* = 0.000 for males). The multiple linear regression model was performed using ferritin/GGT as well as residence, age, TC, LDL-C, UA, UACR, education, income, regular recreational physical activity, heavy drinking, heavy smoking, HBsAg, anti-HCV, AST, and ALT as the independent variables and serum GGT/ferritin as the dependent variable. Stepwise linear regression analysis further confirmed that GGT was independently correlated with ferritin in both genders and showed different independent correlations in different genders (*p* < 0.05). In females, when GGT was used as dependent variable, the standard *β* was calculated as 0.156 (*R* ^2^ = 0.218, *p* = 0.041); when ferritin was used as dependent variable, the standard *β* was calculated as 0.150 (*R* ^2^ = 0.398, *p* = 0.000). In males, when GGT was used as dependent variable, the standard *β* was calculated as 0.205 (*R* ^2^ = 0.262, *p* = 0.007); when ferritin was used as dependent variable, the standard *β* was calculated as 0.170 (*R* ^2^ = 0.272, *p* = 0.024).

### 3.3. Clinical and Laboratory Characteristics of Subjects by GGT and Ferritin Levels

As described earlier, subjects in each gender were divided into three groups according to their serum levels of GGT and ferritin. The detailed clinical and laboratory findings by group and gender are presented in Table 2. Female subjects in Groups 2 and 3 exhibited higher BMI, waist circumference, SP, DP, 2h PG, TG, and HOMA-IR compared to Group 1, with the highest values in Group 3 (*p* < 0.05), and higher FPG in Group 3 compared with Group 1 (*p* < 0.05). Male subjects exhibited higher BMI, waist circumference, DP, and TG from Group 1 to Group 3 (*p* < 0.05) and higher SP, FPG, 2h PG, HDL-C, and HOMA-IR in Group 3 compared with Group 1 (*p* < 0.05).

### 3.4. Risk of Metabolic Syndrome by GGT and Ferritin Levels

Multiple logistic regression tests showed that the risk of metabolic syndrome increased 3.7-fold (95% CI, 2.3–5.7; *p* = 0.000) for Group 3 and 2.8-fold (95% CI, 1.8–4.4; *p* = 0.000) for Group 2 compared to Group 1 according to the revised NCEP-ATP III criteria, and 9.6-fold (95% CI, 5.4–17.4; *p* = 0.000) for Group 3 and 3.1-fold (95% CI, 1.6–5.7; *p* = 0.000) for Group 2 compared to Group 1 according to the CDS criteria. After adjustment for gender, the risk of metabolic syndrome increased 6.1-fold (95% CI, 3.7–9.9; *p* = 0.000) for Group 3 and 3.2-fold (95% CI, 2.0–5.1; *p* = 0.000) for Group 2 compared to Group 1 according to the revised NCEP-ATP III criteria, and 10.7-fold (95% CI, 5.9–19.5; *p* = 0.000) for Group 3 and 3.1-fold (95% CI, 1.7–5.8; *p* = 0.000) for Group 2 compared to Group 1 according to the CDS criteria.

Multiple logistic regression analyses were conducted in both genders separately. The risk of metabolic syndrome increased 7.9-fold (95% CI, 4.6–13.5) according to the revised NCEP-ATP III criteria and 10.3-fold (95% CI, 5.0–21.5) according to the CDS criteria for Group 3 compared to Group 1 in female subjects (*p* = 0.000). This was independent of subjects' residence, age, TC, LDL-C, UA, UACR, recreational physical activity, income, education, heavy drinking, heavy smoking, HBsAg, anti-HCV, ALT, or AST (OR [95% CI] 3.1 [1.7–5.9], *p* = 0.011 according to the NCEP-ATP III criteria; OR [95% CI] 3.5 [1.6–8.2], *p* = 0.017 according to the CDS criteria). However, the risk of metabolic syndrome did not significantly increase for Group 3 compared to Group 1 in male subjects (*p* > 0.05) (Table 3).

### 3.5. Components of the Metabolic Syndrome Risks between Groups in Women

Given the strong association of GGT and ferritin levels with risk of metabolic syndrome in women indicated by our data, we went on to evaluate the likelihood of possessing each of the diagnostic components of the metabolic syndrome (as defined by NCEP-ATP III and CDS) for these groups in female subjects. Specifically, we assessed the association of GGT and ferritin with overweight or obesity, abdominal obesity, hypertriglyceridemia, hypertension, hyperglycemia, and insulin resistance. These results are presented in Table 4. The risk of possessing metabolic syndrome components was significantly higher overall in Group 3 compared with Group 1 (*p* < 0.05), although the risk of low HDL-C did not significantly increase in Group 3 (*p* > 0.05) (Table 4). The statistical significance of these findings persisted even after adjustment for subjects' residence, age, TC, LDL-C, UA, UACR, education, income, recreational physical activity, heavy drinking, heavy smoking, HBsAg, anti-HCV, AST, and ALT conditions (*p* < 0.05) (Table 4).

## 4. Discussion and Conclusions

The Yi people are the seventh largest of the 55 ethnic minority groups officially recognized by the People's Republic of China. They live primarily in rural areas of Southwest China, usually in mountainous regions. From the standpoint of conducting population-based studies, these particular demographic characteristics provided this study with the advantage of performing a detailed study within a relatively homogeneous group of people. This study showed, for the first time, the prevalence of the metabolic syndrome in a relatively large population of Liangshan Yi ethnic minority, out of a total population of 2.31 million.

Our study produced several important findings. First, in our study population, the prevalence of the metabolic syndrome was 197 (19.2%) in the overall population, 161 (27.4%) in female and 36 (8.3%) in male, based on the revised NCEP-ATP III criteria, while it was 135 (13.2%) in the overall population, 79 (13.4%) in female and 56 (12.8%) in male by the CDS definition. Second, serum GGT and ferritin concentrations were significantly higher in subjects with metabolic syndrome compared to those without metabolic syndrome in both genders. Third, serum GGT and ferritin were independently significantly correlated with each other in both genders. Finally, the risks of having metabolic syndrome and each of its diagnostic components (overweight or obesity, abdominal obesity, hypertriglyceridemia, hypertension, hyperglycemia, and insulin resistance) were significantly increased in those female subjects who had elevated GGT and ferritin levels. All these observations remained statistically significant after adjustment for possible confounders.

In clinical practice, an elevated serum GGT level is conventionally interpreted as a marker of alcohol abuse and liver dysfunction. It is still controversial whether elevated GGT or elevated ferritin is a cause or a consequence of the metabolic syndrome. A growing body of evidence suggests that GGT may serve as a predictor of type 2 diabetes and the metabolic syndrome [9–11]. Prospective studies have also found that increased serum ferritin is associated with the development of the metabolic syndrome [13, 14]. In our present study, serum GGT and ferritin were found to be significantly correlated with each other. Multiple regression analysis revealed that the risks of the metabolic syndrome and its components increased significantly in subjects with elevated levels of GGT and ferritin after adjustment for possible confounders, which further confirmed their synergistic associations with the metabolic syndrome and its constituent abnormalities.

From a mechanistic standpoint, GGT and ferritin are both recognized to be involved in oxidative stress, lipid peroxidation, and mitochondrial dysfunction. GGT is the principal enzyme that influences the extracellular hydrolysis of glutathione (GSH). Interestingly, ferritin plays an important role in the catalytic activities of GGT. The reactive products originating from GGT-mediated cleavage of GSH may cause the reduction of ferric iron to ferrous iron. Elevated levels of GGT and ferritin then result in increased production of reactive oxygen species (ROS), aggravating oxidative stress and leading to peroxidation of lipids by highly reactive free radicals. The adverse effects of ferritin overload and increased GGT mutually reinforce each other, ultimately leading to tissue injury [6–8, 18]. Our results are compatible with these findings, and the mechanisms need to be verified and further investigated.

Prior studies have identified regional and sexual variation in the demographics of metabolic syndrome [17, 19–21]. The causes of this observed sexual dimorphism in metabolic syndrome are not well understood. In our study, the significant association between GGT and ferritin and the metabolic syndrome was found in females but not in males. This difference might be attributed to the larger amount of alcohol consumption by males versus females (*p* < 0.05), which could affect GGT levels and distort the ability of GGT levels to reflect oxidative stress. The effect of alcohol consumption on insulin resistance and the metabolic syndrome is considered to be relative to the amount of alcohol intake and patterns and multiple other factors. Whether alcohol consumption is protective or is a risk factor for the metabolic syndrome and/or its components remains controversial in recent studies [22–25]. Furthermore, in this study, the percentage of individuals with the metabolic syndrome was higher in females than in male subjects (according to the revised NCEP ATP-III criteria; *p* < 0.01), though the male population consumed larger amounts of alcohol overall. Further studies should be carried out to understand whether the sexual dimorphism disappears after controlling for the effect of alcohol consumption.

Interestingly, there were a higher proportion of female subjects with larger waist circumference than males in this cohort. This might explain why the prevalence of the metabolic syndrome (according to the revised NCEP-ATP III criteria) is much higher in females than in males. Also, the number of subjects with larger waist circumference was higher than those with larger BMI, especially in females, and this might be one of the reasons why the prevalence of the metabolic syndrome according to the revised NCEP ATP-III criteria was higher than the prevalence according to CDS criteria. Thus, it is important to note that the prevalence of the metabolic syndrome may vary in part due to different metabolic syndrome definitions being applied.

Since the incidence of the metabolic syndrome is increasing rapidly worldwide and carries with it high morbidity and mortality, finding predictive biomarkers may help to improve prevention, treatment, and prognosis for patients. The findings from our study suggest that utilization of the common laboratory values serum GGT and ferritin might be useful in clinical practice. In conclusion, this study showed that GGT and ferritin were significantly correlated with each other and increased in subjects with metabolic syndrome in both genders, while elevated levels of both GGT and ferritin were associated with increased risk of the metabolic syndrome only in females. Further mechanistic and clinical studies are needed to determine the potential use of GGT and ferritin as predictive biomarkers of the metabolic syndrome and its related diseases.

## Figures and Tables

**Table 1:** Clinical and laboratory characteristics of subjects with and without metabolic syndrome according to the revised NCEP-ATP III and CDS criteria.

	Revised NCEP-ATP III criteria		CDS criteria	
	MS	No MS	MS	No MS
Female				
*N*	161 (27.4%)	427 (72.6%)	79 (13.4%)	509 (86.6%)
Age	50.3, 11.4	41.9, 13.6^*∗∗*^	49.1, 11.9	43.5, 13.7^*∗∗*^
GGT (U/L)	32.0 (20.0, 58.0)	18.0 (14.0, 28.0)^*∗∗*^	50.0 (28.0, 77.0)	17.0 (13.0, 27.0)^*∗∗*^
Ferritin (*µ*mol/L)	117.7 (63.8, 169.3)	52.0 (27.9, 113.1)^*∗∗*^	121.5 (54.2, 246.0)	61.9 (29.8, 124.8)^*∗∗*^
BMI (kg/m^2^)	24.7, 3.5	21.5, 3.3^*∗∗*^	26.5, 3.3	21.8, 3.3^*∗∗*^
WC (cm)	86.6, 8.7	75.5, 8.6^*∗∗*^	89.5, 8.8	76.9, 9.1^*∗∗*^
SP (mmHg)	121.5, 21.2	108.2, 15.2^*∗∗*^	125.2, 21.8	109.8, 16.6^*∗∗*^
DP (mmHg)	82.8, 12.6	73.7, 11.5^*∗∗*^	85.1, 12.9	74.8, 11.9^*∗∗*^
TG (mmol/L)	2.5, 1.6	1.2, 0.9^*∗∗*^	2.7, 1.9	1.4, 1.0^*∗∗*^
HDL-C (mmol/L)	1.1, 0.4	1.2, 0.3	1.2, 0.5	1.2, 0.3
TC (mmol/L)	4.5, 0.9	4.1, 0.9^*∗∗*^	4.6, 1.0	4.2, 0.9^*∗*^
LDL-C (mmol/L)	2.7, 0.6	2.4, 0.6^*∗∗*^	2.6, 0.6	2.4, 0.6^*∗*^
FPG (mmol/L)	5.3, 1.6	4.7, 0.7^*∗∗*^	5.8, 2.0	4.8, 0.7^*∗∗*^
2 h PG (mmol/L)	8.0, 4.0	5.8, 1.6^*∗∗*^	9.9, 4.4	5.9, 1.8^*∗∗*^
HOMA-IR	1.8 (1.3, 2.6)	1.2 (0.9, 1.7)^*∗∗*^	2.3 (1.5, 3.2)	1.2 (0.9, 1.8)^*∗∗*^
Place (rural, %)	81 (50.3%)	234 (54.8%)	25 (31.6%)	290 (57.0%)^*∗∗*^
UA (mmol/L)	317.6, 83.9	281.4, 66.4^*∗∗*^	345.0, 107.3	283.5, 63.6^*∗∗*^
UACR (mg/mmol)	12.4 (5.8, 30.2)	11.1 (4.8, 20.4)^*∗*^	19.5 (8.6, 49.8)	10.5 (4.8, 20.4)^*∗∗*^
AST (U/L)	36.0, 20.4	31.3, 12.3^*∗*^	37.8, 17.5	31.8, 14.5^*∗*^
ALT (U/L)	46.1, 38.0	33.5, 18.6^*∗∗*^	53.6, 37.0	34.5, 23.6^*∗∗*^
Education (*N*, %)^†^	16 (10.0%)	68 (15.9%)	14 (17.7%)	70 (13.7%)
Income (*N*, %)^‡^	59 (36.6%)	143 (33.5%)	39/40 (49.4%)	163 (32.0%)^*∗*^
Activity (*N*, %)^§^	61 (37.9%)	124 (29.0%)^*∗*^	37 (46.8%)	148 (29.1%)^*∗*^
Heavy smoking (*N*)^¶^	0	0	0	0
Heavy drinking (*N*, %)^††^	13 (8.1%)	33 (7.7%)	11 (13.9%)	35 (6.9%)^*∗*^
HBsAg (*N*, %)	21 (13.0%)	44 (10.3%)	11 (13.9%)	54 (10.6%)
Anti-HCV (*N*, %)	4 (2.5%)	8 (1.9)	2 (2.5%)	10 (2.0%)
Male				
*N*	36 (8.3%)	400 (91.7%)	56 (12.8%)	380 (87.2%)
Age	46.1, 13.5	45.9, 14.8	49.7, 12.5	44.4, 14.9^*∗*^
GGT (U/L)	63.0 (26.0, 128.0)	35.0 (23.0, 59.0)^*∗∗*^	78.0 (44.0, 140.0)	32.0 (21.0, 53.0)^*∗∗*^
Ferritin (*µ*mol/L)	272.9 (175.4, 349.0)	185.9 (135.2, 298.6)^*∗∗*^	332.8 (262.2, 392.6)	174.5 (127.7, 285.5)^*∗∗*^
BMI (kg/m^2^)	24.5, 4.1	22.0, 3.2^*∗∗*^	26.8, 2.7	21.5, 2.9^*∗∗*^
WC (cm)	89.5, 11.6	80.0, 10.5^*∗∗*^	95.0, 9.3	78.6, 9.4^*∗∗*^
SP (mmHg)	122.6, 15.1	113.7, 16.4^*∗*^	128.2, 15.3	112.3, 15.7^*∗∗*^
DP (mmHg)	84.7, 10.2	77.2, 11.5^*∗∗*^	88.4, 10.6	76.2, 10.9^*∗∗*^
TG (mmol/L)	3.3, 4.5	1.5, 1.4^*∗*^	3.4, 3.9	1.4, 1.1^*∗∗*^
HDL-C (mmol/L)	0.9, 0.2	1.1, 0.3^*∗∗*^	1.2, 0.4	1.1, 0.3
TC (mmol/L)	4.4, 1.1	4.2, 0.9	4.8, 0.9	4.1, 0.9^*∗∗*^
LDL-C (mmol/L)	2.7, 0.8	2.5, 0.6	2.8, 0.7	2.4, 0.6^*∗∗*^
FPG (mmol/L)	6.0, 1.7	5.9, 18.4	5.7, 2.3	5.9, 19
2 h PG (mmol/L)	8.7, 4.2	6.0, 2.7^*∗*^	9.8, 3.6	5.7, 2.4^*∗∗*^
HOMA-IR	1.8 (1.2, 3.2)	1.3 (0.9, 1.8)^*∗*^	2.0 (1.3, 3.3)	1.2 (0.9, 1.8)^*∗*^
Place (rural, %)	22 (61.1%)	261 (65.3%)	12 (21.4%)	271 (71.3%)^*∗∗*^
UA (mmol/L)	402.5, 95.3	372.3, 89.2	441.0, 82.9	365.0, 86.9^*∗∗*^
UACR (mg/mmol)	26.6 (7.9, 77.3)	9.9 (4.0, 19.7)^*∗*^	20.5 (9.4, 60.3)	9.5 (3.7, 19.2)^*∗*^
AST (U/L)	46.2, 31.7	38.9, 40.4	43.7, 27.1	38.8, 41.5
ALT (U/L)	61.2, 40.1	49.0, 66.1	65.2, 41.3	47.6, 67.2
Education (*N*, %)	10 (27.8%)	76 (19.0%)	26 (46.4%)	60 (15.8%)^*∗∗*^
Income (*N*, %)	16 (44.4%)	144 (36.0%)	20 (35.7%)	140 (36.8%)
Activity (*N*, %)	15 (41.7%)	127 (31.8%)	13 (23.2%)	129 (33.9%)
Heavy smoking (*N*, %)	6 (16.7%)	19 (4.8%)^*∗*^	5 (8.9%)	20 (5.3%)
Heavy drinking (*N*, %)	21 (58.3%)	201 (50.3%)	30 (53.6%)	192 (50.5%)
HBsAg (*N*, %)	6 (16.7%)	53 (13.3%)	8 (14.3%)	51 (13.4%)
Anti-HCV (*N*, %)	3 (8.3%)	20 (5%)	3 (5.4%)	20 (5.3%)

Data of age, BMI, WC, SP, DP, TG, TC, HDL-C, LDL-C, FPG, 2 h PG, UA, AST, and ALT are mean, standard deviation; data of GGT, ferritin, HOMA-IR, and UACR are median, interquartile range; other data are *N*, %. ^*∗*^ *p* < 0.05 compared with MS subjects; ^*∗∗*^ *p* < 0.01 compared with MS subjects. ^†^Higher level of education as college or higher. ^‡^Higher level of income as annual income more than 5000 renminbi (RMB). ^§^Regular recreational physical activity as moderate or vigorous activity for 30 minutes or more per day at least 3 days a week. ^¶^Smoking 25^+^ cigarettes per day. ^††^Drinking 60^+^ mL of alcohol per day.

MS: metabolic syndrome; BMI: body mass index; WC: waist circumference; SP: systolic pressure; DP: diastolic pressure; TG: triglyceride; HDL-C: high density lipoprotein-cholesterol; TC: total cholesterol; LDL-C: low density lipoprotein-cholesterol; FPG: fasting plasma glucose; PG: postprandial plasma glucose; UA: uric acid; UACR: urinary albumin to creatinine ratio; AST: aspartate aminotransferase; ALT: alanine aminotransferase; HOMA-IR: insulin resistance assessed by homeostasis model assessment; HBsAg: hepatitis B surface antigen; anti-HCV: anti-hepatitis C virus.

**Table 2:** Clinical and laboratory characteristics of subjects in groups defined by serum GGT and ferritin levels.

	Group 1	Group 2	Group 3
Female			
*N*	171 (29.1%)	228 (38.8%)	189 (32.1%)
Age	37.1, 11.6	44.8, 13.8^*∗∗*^	50.6, 11.8^*∗∗*,‡^
GGT (U/L)	12.0 (10.0, 14.0)	18.0 (12.0, 28.0)^*∗∗*^	31.0 (20.0, 54.0)^*∗∗*,†^
Ferritin (*µ*mol/L)	22.0 (15.6, 42.1)	63.4 (33.2, 112.9)^*∗∗*^	119.4 (109.8, 224.5)^*∗∗*,‡^
BMI (kg/m^2^)	20.9, 2.6	22.0, 3.6^*∗∗*^	24.4, 3.9^*∗∗*,‡^
WC (cm)	74.2, 7.3	77.3, 9.4^*∗∗*^	84.4, 10.3^*∗∗*,‡^
SP (mmHg)	105.9, 12.5	111.2, 17.9^*∗*^	118.4, 20.7^*∗∗*,†^
DP (mmHg)	72.4, 10.3	76.2, 12.6^*∗*^	79.8, 13.4^*∗∗*,†^
TG (mmol/L)	1.1, 0.6	1.6, 1.2^*∗*^	2.1, 1.7^*∗∗*,†^
HDL-C (mmol/L)	1.2, 0.3	1.2, 0.4	1.3, 0.4
TC (mmol/L)	3.9, 0.7	4.2, 0.9^*∗∗*^	4.6, 1.1^*∗∗*^
LDL-C (mmol/L)	2.3, 0.5	2.5, 0.7^*∗*^	2.7, 0.7^*∗∗*,†^
FPG (mmol/L)	4.7, 0.6	5.0, 1.2^*∗*^	5.1, 1.1^*∗∗*^
2 h PG (mmol/L)	5.5, 1.5	6.4, 2.6^*∗∗*^	7.8, 3.4^*∗∗*,†^
HOMA-IR	1.2 (0.9, 1.8)	1.3 (0.9, 2.0)^*∗*^	1.6 (1.1, 2.5)^*∗∗*,‡^
Place (rural, %)	85 (49.7%)	130 (57.0%)	100 (52.9%)
UA (mmol/L)	270.3, 57.1	288.6, 69.9^*∗*^	315.4, 84.6^*∗∗*,†^
UACR (mg/mmol)	8.7 (3.3, 15.0)	11.6 (5.6, 22.3)	15.0 (6.4, 28.2)^*∗*^
AST (U/L)	27.5, 8.4	32.2, 11.6^*∗*^	38.3, 21.3^*∗∗*,‡^
ALT (U/L)	27.1, 12.2	35.0, 18.8^*∗∗*^	49.3, 37.3^*∗∗*,‡^
Education (*N*, %)	36 (21.1%)	31 (13.6%)	17 (9.0%)^*∗*^
Income (*N*, %)	66 (38.6%)	76 (33.3%)	60 (31.7%)
Activity (*N*, %)	40 (23.4%)	76 (33.3%)^*∗*^	69 (36.5%)^*∗*^
Heavy smoking (*N*)	0	0	0
Heavy drinking (*N*, %)	13 (7.6%)	14 (6.1%)	19 (10.1%)
HBsAg (*N*, %)	23 (13.5%)	25 (11.0%)	17 (9.0%)
Anti-HCV (*N*, %)	4 (2.3%)	4 (1.8%)	4 (2.1%)
Male			
*N*	139 (31.9%)	156 (35.8%)	141 (32.3%)
Age	43.0, 16.3	46.1, 14.5	46.3, 13.0
GGT (U/L)	17.0 (13.0, 23.0)	29.0 (22.0, 37.0)^*∗∗*^	67.0 (43.0, 126.0)^*∗∗*,‡^
Ferritin (*µ*mol/L)	107.1 (74.0, 145.8)	188.9 (135.9, 239.1)^*∗∗*^	287.7 (230.5, 353.4)^*∗∗*,‡^
BMI (kg/m^2^)	20.2, 2.2	22.1, 3.0^*∗∗*^	24.2, 3.6^*∗∗*,‡^
WC (cm)	74.0, 7.0	80.2, 9.4^*∗∗*^	88.0, 10.9^*∗∗*,‡^
SP (mmHg)	109.6, 14.2	114.5, 15.8^*∗*^	119.0, 18.0^*∗∗*^
DP (mmHg)	74.2, 10.9	77.3, 10.2^*∗*^	81.8, 12.2^*∗∗*,†^
TG (mmol/L)	1.2, 0.7	1.4, 1.0^*∗*^	2.5, 2.8^*∗∗*,‡^
HDL-C (mmol/L)	1.1, 0.3	1.2, 0.3^*∗*^	1.2, 0.3^*∗∗*^
TC (mmol/L)	3.8, 0.7	4.2, 0.8^*∗∗*^	4.7, 1.0^*∗∗*,‡^
LDL-C (mmol/L)	2.3, 0.6	2.5, 0.6^*∗*^	2.8, 0.7^*∗∗*,‡^
FPG (mmol/L)	4.9, 1.1	5.0, 0.9	5.3, 1.6^*∗*^
2 h PG (mmol/L)	5.7, 2.5	5.9, 2.7	7.1, 3.4^*∗∗*,†^
HOMA-IR	1.2 (0.9, 1.6)	1.3 (0.9, 1.8)	1.4 (1.0, 2.1)^†^
Place (rural, %)	90 (64.7%)	102 (65.4%)	91 (64.5%)
UA (mmol/L)	355.4, 82.5	353.8, 83.3	412.4, 91.3^*∗∗*,‡^
UACR (mg/mmol)	8.6 (3.1, 18.4)	9.9 (3.7, 19.0)	11.2 (4.6, 27.5)^*∗∗*^
AST (U/L)	33.7, 12.9	33.9, 16.5	50.1, 63.7^*∗*,†^
ALT (U/L)	35.8, 18.5	40.6, 27.4	72.3, 101.8^*∗∗*,†^
Education (*N*, %)	23 (16.5%)	35 (22.4%)	28 (19.9%)
Income (*N*, %)	36 (25.9%)	61 (39.1%)^*∗*^	63 (44.7%)^*∗*^
Activity (*N*, %)	41 (29.5%)	48 (30.8%)	53 (37.6%)
Heavy smoking (*N*, %)	5 (3.6%)	10 (6.4%)	10 (7.1%)
Heavy drinking (*N*, %)	60 (43.2%)	81 (51.9%)	81 (57.4%)
HBsAg (*N*, %)	21 (15.1%)	23 (14.7%)	15 (10.6%)
Anti-HCV (*N*, %)	6 (4.3%)	8 (5.1%)	9 (6.4%)

Data of age, BMI, WC, SP, DP, TG, TC, HDL-C, LDL-C, FPG, 2 h PG, UA, AST, and ALT are mean, standard deviation; data of GGT, ferritin, HOMA-IR, and UACR are median, interquartile range; other data are *N*, %. ^*∗*^ *p* < 0.05 compared with Group 1; ^*∗∗*^ *p* < 0.01 compared with Group 1; ^†^ *p* < 0.05 compared with Group 2; ^‡^ *p* < 0.01 compared with Group 2.

**Table 3:** Odds ratios (95% CI) and *p* values for the metabolic syndrome by serum GGT and ferritin levels and by diagnostic criteria.

				Group 1	Group 2	Group 3
NCEP criteria	Female	Model 1^†^	OR (95% CI)	1	4.0 (2.5–6.4)	7.9 (4.6–13.5)
			*p*	—	0.000	0.000
		Model 2^‡^	OR (95% CI)	1	2.5 (1.5–4.3)	3.1 (1.7–5.9)
			*p*	—	0.012	0.011
	Male	Model 1^†^	OR (95% CI)	1	1.1 (0.4–3.2)	2.8 (1.1–6.8)
			*p*	—	0.848	0.027
		Model 2^‡^	OR (95% CI)	1	1.0 (0.2–4.3)	3.4 (0.9–12.4)
			*p*	—	0.996	0.060

CDS criteria	Female	Model 1^†^	OR (95% CI)	1	3.8 (1.8–7.8)	10.3 (5.0–21.5)
			*p*	—	0.001	0.000
		Model 2^‡^	OR (95% CI)	1	2.2 (0.9–4.9)	3.5 (1.6–8.2)
			*p*	—	0.087	0.017
	Male	Model 1^†^	OR (95% CI)	1	2.1 (0.7–6.3)	10.2 (3.9–26.4)
			*p*	—	0.205	0.000
		Model 2^‡^	OR (95% CI)	1	1.0 (0.3–4.1)	2.8 (0.8–10.0)
			*p*	—	0.905	0.095

Data are ORs (95% CI) and *p* values. ^†^Before adjustment. ^‡^After adjustment for place of residence, age, TC, LDL-C, UA, UACR, education, income, regular recreational physical activity, heavy drinking, heavy smoking, HBsAg, anti-HCV, AST, and ALT.

**Table 4:** Odds ratios (95% CI) and *p* values for components of the metabolic syndrome by GGT and ferritin levels in women.

			Group 1	Group 2	Group 3
	Model 1^†^				

MS (NCEP)	Abdominal obesity	OR (95% CI)	1	2.8 (1.9–4.2)	7.9 (4.7–13.1)
		*p*	—	0.006	0.000
	Hypertension	OR (95% CI)	1	1.9 (1.1–3.3)	5.8 (3.3–10.2)
		*p*	—	0.033	0.000
	High fasting glucose	OR (95% CI)	1	2.0 (0.9–4.8)	5.2 (2.2–12.2)
		*p*	—	0.061	0.000
	Hypertriglyceridemia	OR (95% CI)	1	2.9 (1.8–4.5)	5.9 (3.5–9.8)
		*p*	—	0.004	0.000
	Low HDL-C	OR (95% CI)	1	1.0 (0.7–1.4)	0.7 (0.4–1.0)
		*p*	—	0.925	0.170

MS (CDS)	Overweight or obesity	OR (95% CI)	1	3.9 (2.4–6.4)	6.3 (3.6–10.9)
		*p*	—	0.000	0.000
	Dyslipidemia	OR (95% CI)	1	0.9 (0.4–2.2)	1.5 (0.7–3.5)
		*p*	—	0.281	0.072
	Hypertension	OR (95% CI)	1	1.6 (1.0–2.6)	3.3 (2.1–5.3)
		*p*	—	0.038	0.001
	Hyperglycemia	OR (95% CI)	1	2.1 (1.2–4.0)	4.9 (2.5–9.7)
		*p*	—	0.019	0.000

Insulin resistance (WHO)		OR (95% CI)	1	1.9 (1.3–2.7)	2.2 (1.4–3.5)
		*p*	—	0.021	0.018

	Model 2^‡^				

MS (NCEP)	Abdominal obesity	OR (95% CI)	1	1.8 (1.2–2.8)	2.8 (1.5–5.1)
		*p*	—	0.025	0.017
	Hypertension	OR (95% CI)	1	1.3 (0.7–2.4)	2.9 (1.5–6.0)
		*p*	—	0.384	0.015
	High fasting glucose	OR (95% CI)	1	1.5 (0.6–3.8)	3.6 (1.6–9.9)
		*p*	—	0.477	0.010
	Hypertriglyceridemia	OR (95% CI)	1	1.8 (1.1–3.1)	2.6 (1.4–5.2)
		*p*	—	0.034	0.021
	Low HDL-C	OR (95% CI)	1	0.9 (0.5–1.6)	0.8 (0.4–1.8)
		*p*	—	0.679	0.653

MS (CDS)	Overweight or obesity	OR (95% CI)	1	2.3 (1.1–4.9)	2.8 (1.7–5.2)
		*p*	—	0.023	0.015
	Dyslipidemia	OR (95% CI)	1	0.8 (0.3–2.3)	0.8 (0.4–2.4)
		*p*	—	0.946	0.878
	Hypertension	OR (95% CI)	1	1.4 (0.8–2.4)	2.3 (1.3–4.3)
		*p*	—	0.211	0.031
	Hyperglycemia	OR (95% CI)	1	1.5 (0.7–3.2)	2.8 (1.2–7.5)
		*p*	—	0.162	0.018

Insulin resistance (WHO)		OR (95% CI)	1	1.7 (1.1–2.6)	1.7 (1.3–3.4)
		*p*	—	0.032	0.028

Data are ORs (95% CI) and *p* values. ^†^Before adjustment. ^‡^After adjustment for place of residence, age, TC, LDL-C, UA, UACR, education, income, recreational physical activity, heavy drinking, heavy smoking, HBsAg, anti-HCV, AST, and ALT.
